# Digital Twin Technology in Resolving Polycystic Ovary Syndrome and Improving Metabolic Health: A Comprehensive Case Study

**DOI:** 10.1016/j.aace.2024.11.004

**Published:** 2024-11-22

**Authors:** Paramesh Shamanna, Anuj Maheshwari, Ashok Keshavamurthy, Sanjay Bhat, Abhijit Kulkarni, Shivakumar R, Kumar K, Mukulesh Gupta, Mohamed Thajudeen, Ranjita Kulkarni, Shashikiran Patil, Shashank Joshi

**Affiliations:** 1 Bangalore Diabetes Centre, Bangalore, Karnataka, India; 2 Shri Hari Kamal Diabetes and Heart Clinic, Lucknow, Uttar Pradesh, India; 3 Chandana Clinic, Bangalore, Karnataka, India; 4 Department of Cardiology, Mukambika Heart Care, Bangalore, Karnataka, India; 5 Department of Cardiology, South End Speciality Clinics, Bangalore, Karnataka, India; 6 Udyaan Health Care, Lucknow, Uttar Pradesh, India; 7 Twin Health, Mountain View, California; 8 Department of Diabetology and Endocrinology, Lilavati Hospital and Research center, Mumbai, India

**Keywords:** polycystic ovary syndrome (PCOS), Digital Twin technology, insulin resistance, metabolic health, personalized medicine

## Abstract

**Background:**

Clinical manifestations of polycystic ovary syndrome (PCOS) are heterogeneous, with hallmarks including anovulation, androgen excess, and insulin resistance.

**Case Report:**

A 38-year-old female with typical PCOS features presented with hypertension, obesity, and elevated fasting and postprandial insulin levels. She was enrolled in the Digital Twin (DT) platform, which uses artificial intelligence and Internet of Things to deliver personalized nutrition by predicting postprandial glucose responses and suggesting alternative foods with lower postprandial glucose response through a mobile app. After 360 days, significant improvements were observed. Weight decreased from 65.4 kg to 57.3 kg (−12.4%); body mass index lowered from 26.2 to 22.96 (−12.4%); Waist circumference reduced from 104 cm to 86.3 cm (−17.0%); clinic systolic blood pressure/diastolic blood pressure reduced from 144/93 to 102/80 mmHg (−29.17%/-13.98%); fasting insulin dropped from 27.6 to 15.5 μIU/mL (−43.8%); postprandial insulin decreased from 182.4 to 23.8 μIU/mL (−87.0%); Homeostatic Model Assessment of Insulin Resistance reduced from 6.47 to 3.48 (−46.2%); estimated glomerular filteration rate improved from 116 to 128 mL/min/1.73m2 (+10.3%); urine microalbumin creatinine ratio decreased from 596 to 73 mg/g (−87.8%). Ultrasound showed reduced ovarian volume and improved fatty liver infiltration, while computed tomography scan revealed significant reductions in epicardial (21.8%), pericardial (69.9%), and visceral fat (44.4%).

**Discussion:**

This case shows the effective use of DT technology for managing PCOS, significantly improving weight, body mass index, insulin, blood pressure, and lipid profile. It supports the potential of artificial intelligence-driven, personalized interventions in chronic disease management.

**Conclusion:**

This case highlights the potential of DT technology in managing PCOS, showing significant metabolic and reproductive improvements, suggesting promising future research directions.

**Highlights**

• Digital Twin technology improved metabolic health in PCOS over 360 days
• AI-driven personalized nutrition reduced insulin resistance significantly
• The intervention achieved marked improvements in ovarian morphology
• IoT enabled real-time dietary tracking and personalized health insights
• Digital Twin technology effectively reduced epicardial and pericardial fat volumes

**Clinical Relevance**

The study demonstrates Digital Twin technology's potential in managing polycystic ovary syndrome, significantly improving metabolic health and ovarian morphology through personalized, artificial intelligence-driven interventions.

## Introduction

Polycystic ovary syndrome (PCOS) is a complex endocrine disorder marked by anovulation, hyperandrogenism, and insulin resistance, predisposing individuals to metabolic comorbidities like obesity, type 2 diabetes, and hypertension.^1^^,^^2^ The severity of menstrual dysfunction, often linked to hyperinsulinemia and glycemic abnormalities, can serve as a proxy for metabolic dysfunction in PCOS.^3^ Although lifestyle interventions, such as diet and exercise, are first-line treatments, adherence challenges often lead to suboptimal outcomes, particularly in reproductive health.^4^ Medical treatments like combined oral contraceptives and metformin manage symptoms but have limited impact on fertility, highlighting the need for personalized PCOS management strategies.^5^

Digital Twin (DT) technology,^6^, ^7^, ^8^ using artificial intelligence (AI) and Internet of Things (IoT), predicts personalized postprandial glucose responses (PPGRs) to reduce dysglycemia, hyperinsulinemia, and hyperandrogenism in PCOS, while promoting sustained symptom relief through real-time, personalized recommendations.

This case report showcases the potential of DT technology to transform PCOS management by delivering personalized treatment strategies that significantly improve metabolic health and ovarian morphology, promising better patient outcomes in endocrinology. This example sets the stage for future research and clinical practice in digital health solutions for complex endocrine disorders.

## Case Presentation

### Patient Presentation

A 38-year-old female with a history of PCOS presented with irregular menstrual cycles, unexplained weight gain, and difficulty managing blood pressure. The patient had a family history of type 2 diabetes and cardiovascular disease, increasing her risk of metabolic syndrome. Her body mass index (BMI) was 26.2, indicating overweight status. Clinical examination revealed signs of androgen excess, such as hirsutism, along with obesity. The patient was treatment-naïve before starting the intervention.

## Clinical and Laboratory Evaluations

Baseline tests showed:

• Fasting insulin: 27.6 μIU/mL (normal: 2.6-24.9 μIU/mL), indicating hyperinsulinemia.
• Postprandial insulin: 182.4 μIU/mL, confirming insulin resistance.
• Homeostatic Model Assessment of Insulin Resistance: 6.47 (normal <2.5), suggestive of significant insulin resistance.
• Fasting glucose: 98 mg/dL (normal: 70-99 mg/dL).
• Lipid profile: Elevated low-density lipoprotein at 160 mg/dL (normal <100 mg/dL for high-risk individuals) and reduced high-density lipoprotein at 40 mg/dL (normal >50 mg/dL).

## Imaging Findings

• Pelvic ultrasound:
  • Right ovary measured 2.8 × 1.6 cm and left ovary 3.4 × 2.4 cm, both containing multiple follicles consistent with PCOS.
• Abdominal ultrasound: Showed diffuse fatty liver infiltration.

### Treatment and Monitoring

The patient provided informed consent, and all data were managed according to privacy regulations, as she was enrolled in a DT technology platform that integrated AI and IoT for personalized nutrition and health recommendations via a mobile app.

### Intervention

**Fig. 1:**
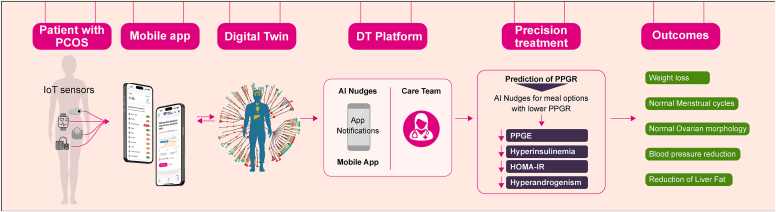
Digital Twin technology workflow for personalized PCOS management. This figure shows the DT technology workflow for personalized PCOS management. Data from IoT sensors — CGM (Abbott FreeStyle Libre Pro), a sensor watch (Fitbit Charge 2), a BP monitor (TAIDOC TD-3140), and a Powermax BCA-130 bluetooth smart scale are transmitted to a mobile app. This data is processed by the DT platform to predict PPGR and deliver personalized recommendations aimed at reducing PPGE, hyperinsulinemia, HOMA-IR, and hyperandrogenism. The outcomes include weight loss, regular menstrual cycles, improved ovarian morphology, reduced blood pressure, and decreased liver fat. Abbreviations: *BP* = blood pressure; *CGM* = continuous glucose monitoring; *DT* = Digital Twin; *HOMA-IR* = Homeostatic Model Assessment of Insulin Resistance; *IoT* = Internet of Things; *PCOS* = polycystic ovary syndrome; *PPGE* = postprandial glycemic excursion; *PPGR* = postprandial glucose response.

Women with PCOS and normal glucose tolerance have changes in postprandial glycemic excursions.^9^ DT technology (Fig. 1), powered by machine learning (ML) algorithms CatBoostRegressor and Random Forest models, integrated data from Continuous Glucose Monitoring (CGM; Abbott FreeStyle Libre Pro), sensor watch (Fitbit Charge 2), blood pressure meter (TAIDOC TD-3140), and Powermax BCA-130 bluetooth smart scale, and dietary inputs from mobile apps. This advanced approach enabled the prediction of personalized PPGR to individual meals, dynamically suggesting alternative meal options tailored to the individual. By effectively minimizing postprandial glucose spikes, this technology helped to reduce dysglycemia, a condition that can occur even in nondiabetic PCOS patients. Scientific evidence suggests that dysglycemia contributes to hyperinsulinemia, a key driver of hyperandrogenism and other PCOS manifestations.^2^ By mitigating postprandial glycemic excursion, DT technology reduced hyperinsulinemia, which in turn lowered systemic inflammation—a factor implicated in the pathophysiology of PCOS. Lowering these metabolic disruptions improved insulin sensitivity and reduced androgen levels, alleviating symptoms such as irregular menstrual cycles, hirsutism, and improving ovarian morphology. The DT platform used dynamic nudges via the mobile app, tailored to real-time data and ML models, to guide healthier lifestyle choices in diet, activity, and sleep. Remote coaching reinforced these AI-driven recommendations, enhancing patient engagement and adherence. Over time, the ML algorithms adapt to the patient’s behavior, refining strategies to optimize outcomes. This integration of personalized behavioral modifications is crucial to the DT approach, promoting sustained lifestyle changes and improved PCOS management.

Although not indicated for PCOS, CGM was used for DT development. Baseline CGM showed an estimated hemoglobin A1c (eA1c) of 5.77, Time-In-Range (TIR) of 95.8%, and Time-Below-Range-1 (TBR-1) of 4.2%. Metformin 500 mg twice daily was started along with the intervention and eA1c dropped to 4.65, TIR to 92.7%, and TBR-1 increased to 7.3% in 2 weeks. Metformin was then reduced to 500 mg once daily, leading to an eA1c of 5.1, TIR of 93%, and TBR-1 of 6.3% after another week. Due to persistently high TBR-1, metformin was stopped on day 21. By day 180, TIR improved to 97.4% and TBR-1% to 2.6%; by day 360, TIR was 98.5% and TBR-1 was 1.5%.

The patient's calorie intake dropped from 2151 Kcal to 1680 Kcal at D180 and 1816 Kcal at D360. Carbohydrates decreased from 330 g/d to 208 g/d at D180 and 225 g/d at D360. These reductions occurred spontaneously due to AI recommendations, increasing vegetable and protein intake, enhancing satiety. Protein rose from 39 g/d to 54 g/d at D180 and 58 g/d at D360, while fat intake slightly shifted from 75 g/d to 70 g/d at D180 and 76 g/d at D360. Total daily PPGR fell from 1120 mg/dl/d to 450 mg/dl/d at D180 and 486 mg/dl/d at D360. The glycemic index dropped from 52 to 42 at D180 and 45 at D360, while glycemic load decreased from 146 to 88 at D180 and 95 at D360. Daily energy expenditure was 1549 Kcal at baseline, 1903 Kcal at D180, and 1867 Kcal at D360.

## Outcomes After 360 Days

**Fig. 2:**
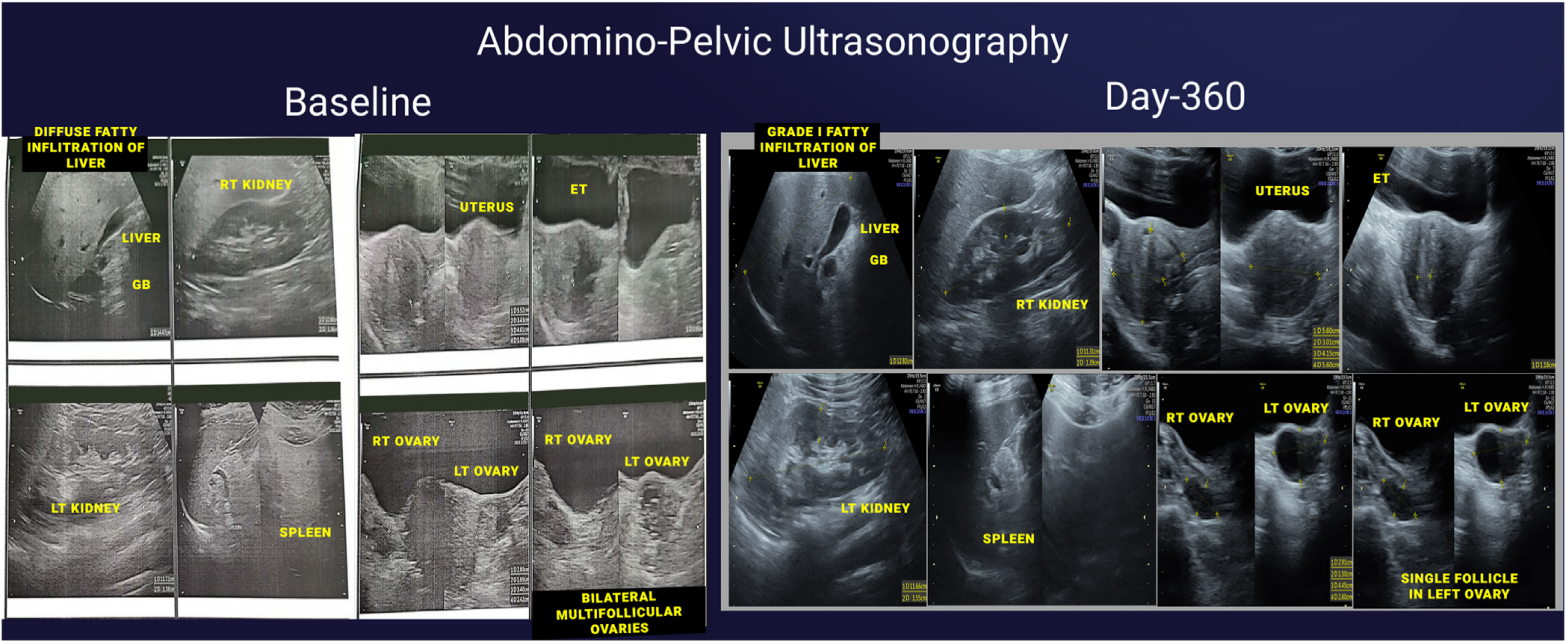
Abdominopelvic ultrasonography between baseline and day-360 baseline ultrasound revealed normal-sized ovaries with multiple follicles, consistent with PCOS: *right* ovary at 2.8 × 1.6 cm, *left* at 3.4 × 2.4 cm; a mildly bulky uterus; and diffuse fatty liver infiltration. After 360 days, *right* ovary measured 2.9 × 1.5 cm, *left* ovary 4.4 × 2.6 cm with a 23 × 19 mm follicle; and improvement to grade I fatty liver infiltration. Abbreviations: *ET* = endometrial thickness; *GB* = gall bladder; *LT* = *left*; *RT* = *right*.
• Computed tomography ccan: Epicardial volume decreased by 21.8% (Fig. 3), pericardial volume by 69.9% (Fig. 4), and visceral fat by 44.4%.

• Menstrual cycles: Regular 28-day cycles.
• Weight and BMI: Reduced to 57.3 kg and 22.96, respectively (12.4% reduction).
• Blood pressure: Home systolic blood pressure/diastolic blood pressure reduced to 108/72 mmHg; clinic systolic blood pressure/diastolic blood pressure to 102/80 mmHg.
• Insulin levels: Fasting insulin decreased to 15.5 μIU/mL; postprandial insulin to 23.8 μIU/mL.
• Lipid profile: Low-density lipoprotein and triglycerides normalized; high-density lipoproteins increased to 55 mg/dL.
• Homeostatic Model Assessment of Insulin Resistance: Improved to 3.48.
• Liver function: Improved to grade I fatty liver infiltration.
• Ovarian morphology: Decreased ovarian volume and follicle count, with no significant cysts (Fig. 2).

**Fig. 3:**
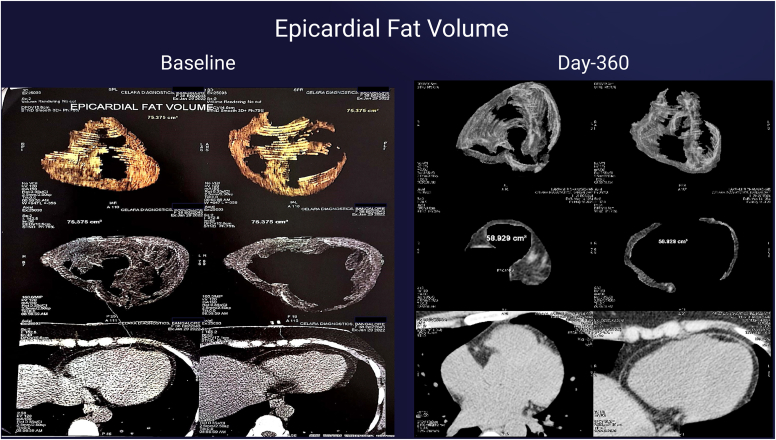
Epicardial fat volume measured by computed tomography (CT) scan at baseline and day 360 epicardial volume decreased from 75.3 cm3 at baseline to 58.92 cm3 at day-360 (21.8% reduction).

**Fig. 4:**
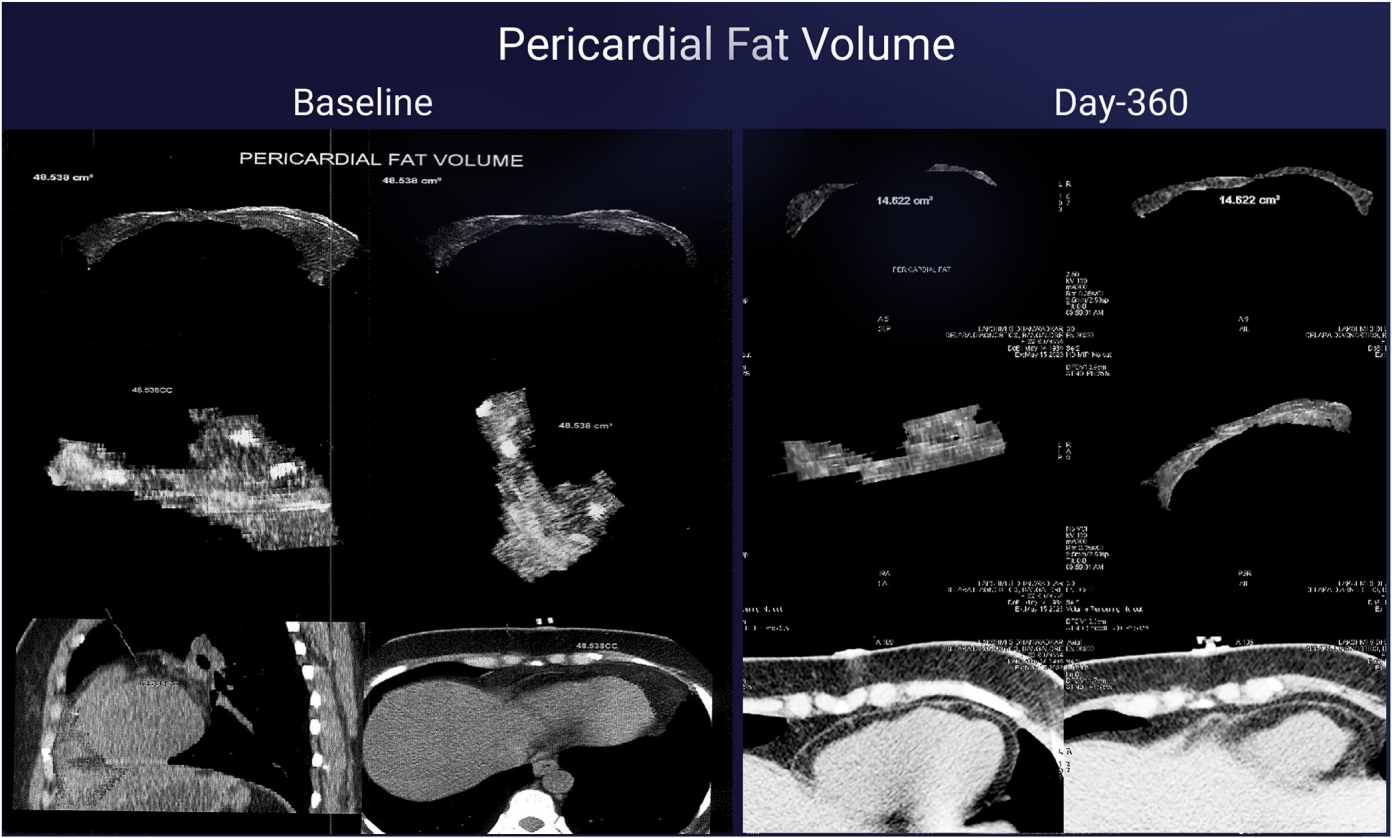
Pericardial fat volume measured by computed tomography (CT) scan at baseline and day 360 pericardial volume from 48.54 cm3 at baseline to 14.6 cm3 at day-360 (69.9% reduction).

## Discussion

This case highlights the potential of DT-guided personalized interventions in significantly improving metabolic health in a 38-year-old PCOS patient, demonstrating the value of digital health technologies in managing PCOS. The patient exhibited typical PCOS symptoms, including menstrual irregularities, hyperandrogenism, hypertension, obesity, and insulin resistance, all of which are known metabolic complications of the disorder.

Management of PCOS typically involves lifestyle changes and medical interventions like combined oral contraceptives and metformin.^5^ However, these treatments often have limited impact on reproductive outcomes and patient adherence is challenging.^4^ This highlights the need for more effective, individualized treatment strategies.

Digital health technologies have increasingly been recognized for their role in enhancing patient outcomes in PCOS.^10^^,^^11^ For instance, the AskPCOS mobile application, designed to deliver evidence-based information and self-management tools, has been shown to address significant gaps in patient knowledge and engagement.^12^

Similarly, the HOPE PCOS digital self-management program demonstrated potential improvements in mental health and well-being, emphasizing the critical role of digital tools in holistic PCOS care.^13^ Our case aligns with these findings, reinforcing the idea that personalized, technology-driven interventions can significantly enhance the management of PCOS, particularly when traditional approaches such as lifestyle modifications and pharmacotherapy show limited efficacy.

In this case, DT technology facilitated a personalized treatment approach by integrating real-time clinical data, lifestyle factors, and patient feedback. Significant improvements in reproductive and metabolic parameters, including reduced weight, BMI, and insulin levels, as well as better blood pressure and lipid profiles, demonstrate the potential of personalized interventions in managing PCOS-related risks.

The case underscores several key points. First, integrating technology into PCOS management can enhance patient engagement and adherence to lifestyle modifications. Second, the use of AI and IoT in health care can create dynamic, personalized care plans, particularly beneficial for conditions like PCOS with varied presentations.

While metformin is recognized for its role in PCOS management, it was only briefly used in this case and discontinued due to concerns with CGM readings. The significant improvements observed over the year—long after metformin was stopped—suggest that the DT intervention played a crucial role in these outcomes. This case contributes to the growing evidence supporting the use of digital health technologies in managing chronic diseases and highlights the potential of DT technology in revolutionizing patient care.

Potential confounders such as baseline metabolic status, medication adherence, and lifestyle factors were carefully managed through comprehensive baseline assessments, real-time data integration, and longitudinal monitoring over 360 days. These steps aimed to ensure that the observed outcomes were primarily attributable to the DT intervention.

However, the single-patient design of this case report limits the generalizability of the findings. While the outcomes are promising, they should be viewed as preliminary. Further research, including larger studies and randomized trials, is needed to validate these findings and determine the broader applicability of DT technology in PCOS management.

In conclusion, this case highlights the potential of DT technology to personalize and optimize PCOS management. While the improvements observed are encouraging, further research is necessary to confirm the efficacy of this approach in a broader patient population.

## Disclosure

Dr Paramesh Shamanna, Dr Ashok Keshavamurthy, Dr Mohamed Thajudeen, Dr Ranjita Kulkarni and Dr Shashikiran Patil are employees of Twin Health Inc. Dr Shashank Joshi is a Scientist at Twin Health Inc., He has received consulting fees from Franco Indian, Biocon, Zydus Cadila, Glenmark, Torrent and Marico. Dr Joshi has received speaker honoraria or has served on the advisory board of MSD, Novo Nordisk, Sanofi, Boehringer Ingelheim, Abbott, Astra Zeneca, Serdia, Alkem, Lupin, Bayer Zydus and USV. Other authors have reported no conflict of interest.
